# Examining fatigue in COPD: development, validity and reliability of a modified version of FACIT-F scale

**DOI:** 10.1186/1477-7525-10-100

**Published:** 2012-08-23

**Authors:** Khaled Al-shair, Hana Muellerova, Janelle Yorke, Stephen I Rennard, Emiel FM Wouters, Nicola A Hanania, Amir Sharafkhaneh, Jørgen Vestbo

**Affiliations:** 1 University of Manchester, Medicines Evaluation Unit, NIHR Translational Research Facility, Manchester Academic Health Sciences Centre, University Hospital of South Manchester Foundation Trust, Wythenshawe, Manchester, UK; 2 Hadhramout University of Science and Technology, College of Medicine, Hadhramout, Yemen; 3 Worldwide Epidemiology, GlaxoSmithKline R&D at Stockley Park, Uxbridge, UK; 4 University of Manchester, School of Nursing and Midwifery, Manchester, UK; 5 University of Nebraska Medical Centre, Omaha, NE, USA; 6 Department of Respiratory Medicine, Maastricht University Medical Centre, Maastricht, The Netherlands; 7 Section of Pulmonary and Critical Medicine, Baylor College of Medicine, Houston, TX, USA; 8 Department of Medicine, Section of Pulmonary, Critical Care and Sleep Medicine, Baylor College of Medicine, Houston, TX, USA; 9 Department of Respiratory Medicine, Odense University Hospital, Odense, Denmark

**Keywords:** Chronic obstructive pulmonary disease, Fatigue, Exercise capacity, Health status

## Abstract

**Introduction:**

Fatigue is a disruptive symptom that inhibits normal functional performance of COPD patients in daily activities. The availability of a short, simple, reliable and valid scale would improve assessment of the characteristics and influence of fatigue in COPD.

**Methods:**

At baseline, 2107 COPD patients from the ECLIPSE cohort completed the Functional Assessment of Chronic Illness Therapy Fatigue (FACIT-F) scale. We used well-structured classic method, the principal components analysis (PCA) and Rasch analysis for structurally examining the 13-item FACIT-F.

**Results:**

Four items were less able to capture fatigue characteristics in COPD and were deleted. PCA was applied to the remaining 9 items of the modified FACIT-F and resulted in three interpretable dimensions: i) general (5 items); ii) functional ability (2 items); and iii) psychosocial fatigue (2 items). The modified FACIT-F had high internal consistency (Cronbach's α = 0.91) and it did not fit a uni-dimensional Rasch model, confirming the prior output from the PCA. The correlations between total score and each dimension were ≥ 0.64 and within dimensions ≥0.43 (p < 0.001 for all).

The original and modified FACIT-F had significant convergent validity; its scores were associated with SGRQ total score (0.69 and 0.7) and mMRC dyspnoea scores (0.48 and 0.47), (p = <0.001 for all). The scale had meaningful discriminating ability in identifying patients with poor exercise performance and more depressive symptoms.

**Conclusion:**

The original and modified FACIT-F are valid and reliable scales in COPD. The modified version is shorter and measures not only total fatigue but also its sub-components in COPD.

## Introduction

Fatigue is a disruptive symptom that inhibits normal functional performance of Chronic Obstructive Pulmonary Disease (COPD) patients in daily activities [1] and considerably impacts on their quality of life [2]. Fatigue together with dyspnoea are the most prominent disabling symptoms in COPD [3,4]. There is growing interest and attention on the substantial impact of fatigue on COPD patients [5,6] and the need of a short, simple, reliable and valid instrument that can improve the assessment of fatigue in COPD.

The Functional Assessment of Chronic Illness Therapy-Fatigue scale (FACIT-F scale) is a 13-item scale [7,8] that was previously employed in cohorts of patients with COPD [5]. However; no study examined its psychometric properties, validity and reliability in this population. Using a well-structured classic framework assessment, principal component analysis (PCA) and Rasch analysis, we examined the psychometric properties of the scale including its underlying structural construct, validity of items and possible dimensionality, followed by examination of its reliability and validity in a large COPD cohort from the Evaluation of COPD Longitudinally to Identify Predictive Surrogate Endpoints (ECLIPSE) study [9]. Overall, we aimed to examine the FACIT-F and create a modified version for COPD patients.

## Methods

The ECLIPSE study (NCT00292552; GlaxoSmithKline study SCO104960) was an observational prospective three-year multi-centre study. It was conducted in 46 centres from 12 countries in accordance with the Declaration of Helsinki and good clinical practice guidelines. A total of 2107 patients were examined at baseline and completed the FACIT-F questionnaire, of these 1621 patients were followed up for 3 years, and completed the questionnaire again at the final study visit. Criteria for enrolment included age between 40–75 years, post-bronchodilator forced expiratory volume in one second (FEV_1_) < 80% of normal predicted, post-bronchodilator FEV_1_/forced vital capacity (FVC) ≤ 0.7 and smoking history ≥ 10 pack-years. At each study visit, patients were clinically stable for at least 4 weeks before each visit. Therefore, during the study conduction many visits had been re-scheduled to meet this criterion. Further details on this study have been previously published [9,10].

### Measurements

The 13-item FACIT-F scale is a self-reported scale, where subjects respond to each item by choosing one of five options (Not at all (4 scores), A little bit (3), Some-what (2), Quite a bit (1), Very much (0) [7,8]. Two items have to be reversely scored, and overall scores of the FACIT-F scale range from 0 to 52, with higher scores signifying less fatigue [7,8]. The scale was originally developed to assess anemia-related fatigue in patients with cancer in a 7 days recall period. It had a test-retest reliability correlation (0.87) and strong internal consistency (Cronbach’s α coefficient 0.95). It had good convergent and discrimination validities; e.g., good correlation with the Piper Fatigue scale (0.75) and Profile of Mood States (0.74), and it differentiated between different levels of haemoglobin in patients with cancer [7].

Study patients also completed: St. George’s Respiratory Questionnaire for COPD patients (SGRQ-C) [11], the modified Medical Research Council (mMRC) Dyspnea scale [12], and the Centre for Epidemiologic Studies of Depression Scale (CES-D) [13]. Lung function tests, and six minute walk distance (6MWD) [14] were measured.

### Statistical analysis

**Figure 1:**
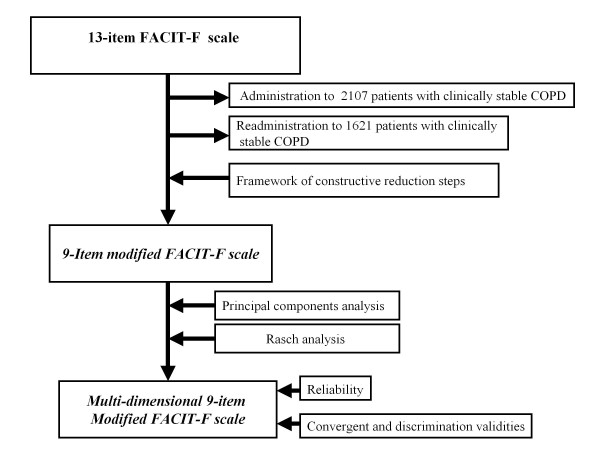
Flow chart illustrating the development of the COPD modified FACT-F scale.

Statistical analyses were conducted in four phases (see Figure 1):

1) Assessment of traditional psychometric properties:

We used a constructive framework that was based on previous experiences [2,15-17] to investigate the presence of:

i). Non-applicable items; i.e., items not applicable to COPD patients’ current life style;

ii). Items showing redundancy of measurement defined by a high correlation with another item;

iii). Items with a correlation coefficient (r^2^ coefficient) <0.5 or >0.9 with all the other items; (r^2^ coefficient is the value of the squared correlation between each item and the total score of all the other items); and

iv). Items with a ‘floor’ or ‘ceiling’ effect (i.e., items that were mostly answered with “Not at all” or “Very much”). Importantly, before any item was removed its clinical importance (content validity) was considered.

2) Principal component analysis (PCA)

An exploratory PCA was performed to examine loadings of the items for each domain using SPSS version 15. Kaiser’s criterion of eigen value >1 and Catell’s scree test were used to determine the number of components [18]. We conducted both orthogonal and oblique rotation techniques and found that oblique (Direct Oblimin) provided the best and easiest interpretation; nevertheless, an orthogonal approach (Varimax) provided a very similar result.

The PCA was separately conducted using two software packages SPSS 15 and Rasch Uni-dimensional Measurement Model 2030 (RUMM) [19,20].

3) Rasch analysis

Rasch analysis is a sophisticated technique which has been widely used to assess the psychometric properties of scales in different fields including respiratory research [11,20]. It is based on the assumption that all items measure a single underlying dimension by examining the fitness and contribution of each item to measure a single construct. It also confirms whether a scale meets the requirements of fundamental measurement, allowing for mathematical investigation for reasons of misfit through the assessment of the parametric properties such as individual person fit and item fit, response thresholds, differential item functioning (DIF), local dependency and person separation index (PSI) [21,22].

Exploratory Rasch analysis was conducted several times before and after removing items from previous steps. Taking advantage of the large sample size, random sampling was used to divide the sample to 4 sub-groups (each >500 patients). This enabled us to conduct the Rasch analysis in the first group and validate the results in the other groups. The Rasch analysis was also performed using the whole sample. RUMM 2030 (Version 5.1 for Windows, RUMM Laboratory, Perth, Western Australia) was used [19,20].

4) Reliability and validity

Reliability of the fatigue scale

We investigated the internal consistency of the original FACIT-F, and the modified version and each of its dimensions using Cronbach’s α coefficient [23]. A correlation ≥ 0.7 was assumed to indicate that questions within a dimension are likely to measure the same construct.

Validity of the fatigue scale

To examine the convergent validity; i.e., the extent to which the scores of a measure are related to scores of other constructs [24], the total score of the original FACIT-F, and the total and dimensional scores of the 9-item modified FACIT-F were correlated with impairment in quality of life and the perception of dyspnoea using SGRQ and mMRC dyspnoea scales. The discriminating validity was also examined comparing the mean fatigue scores between patients with high depressive symptoms and those with low depressive symptoms (using ≥16 scores cut-off of CES-D), and between patients with a poor 6MWT performance (less than 350 metres) or more [25]. Description of additional statistical analysis can be seen in the Additional file 1

## Results

Baseline characteristics and demographic data of the study patients are shown in Table 1.

**Table 1:** Baseline characteristics and demographic of the study population (data are presented as mean (SD) unless otherwise noted)

**Variable**	**Description**
Age (yr)	63.4 (7)
Female (n, %)	732 (35 %)
Current smokers (n, %)	767 (36 %)
Post bronchodilator FEV_1_ (litres)	1.35 ± (0.52)
Post bronchodilator % predicted FEV_1_	48 ± (16)
FEV_1_/FVC (%)	45 ± (12)
GOLD stages II, III, and IV (n, (%))	925 (44 %), 894 (42 %), 292 (14 %)
BMI (kg/m^2^)	26.6 ± (5.7)
6MWD (m)	370.1 ± (121.7)
SGRQ (Total score)	49.9 ± (20.1)
mMRC dyspnoea (Median (IQR))	2 (1 – 2)
CES-D score (Median (IQR))	9 (4 – 16)

FEV_1_: Forced expiratory volume in one second; FVC: forced vital capacity; BMI: body mass index as measured by kilograms over height in meter squared; 6MWD: 6 minutes walk distance; mMRC: the modified Medical Research Council dyspnoea scale; SGRQ: St. George’s Respiratory Questionnaire; CES-D: The Centre for Epidemiologic Studies Depression scale.

1) Assessment of classic psychometric properties:

Using constructive steps of the classic psychometric properties assessment, 4 items were unlikely to efficiently assess fatigue in COPD and these were proposed for deletion as detailed below and in Additional file 1 Table S1:

**“I am too tired to eat”**, 1618 patients (76.8%) responded to this item with “not at all” indicating lack of applicability in COPD with a low squared correlation coefficient, (r^2^ =0.28) and high floor effect.

**“I need help to do my usual activities”,** 1290 patients (61.5%) responded to this item with “not at all”. The mean score has shown moderate high floor effect, further indicating lack of applicability in COPD.

**“I need to sleep during the day”,** we found that 687 patients (32.6%) responded to it with “not at all” indicating relatively lack of applicability in COPD, and it also showed a low r^2^ coefficient, 0.29.

**“I feel tired”** had a high correlation with 2 other items (items 1 and 5) indicating redundancy particularly with item “I feel fatigued”.

2) Principal component analysis

The remaining 9 item FACIT-F was subjected to the PCA. For comparison and to assess advantages of the previous shortening step, we also ran the PCA for the whole 13-item scale ( Additional file 2). The 9 items of FACIT-F were subjected to the PCA using SPSS version 15 and RUMM 2030.

The suitability of data for factor analysis was assessed. Inspection of the correlation matrix revealed the presence of many coefficient correlations of 0.3 and above. The Kaiser-Meyer-Oklin value was 0.9, exceeding the recommended value of 0.6 and Bartlett’s Test of Sphericity reached statistical significance (<0.001), supporting the factorability of the correlation matrix.

PCA revealed the presence of three components. To aid on interpretation of these components, several rotation techniques revealed the presence of a simple structure with three components. Most of the items loading substantially on component 1 describing the general impact of fatigue (general fatigue component), items describing the impact on energetic and functional status loaded on component 2 (functional ability component), and items describing the psychosocial impact of fatigue loaded on component 3 (psychosocial component) as shown in Table 2.

**Table 2:** Pattern and Structure Matrix for PCA with Oblimin Rotation of three factors solution of the 9-item FACIT scale (loading values ≤ 0.3 were not demonstrated)

**Items**	**Components**					
	**Component 1**		**Component 2**		**Component 3**	
	**Pattern**	**Structure**	**Pattern**	**Structure**	**Pattern**	**Structure**
1 I feel weak all over	**0.94**	**0.89**		0.30		0.56
2 I feel fatigued	**0.83**	**0.86**		0.30		0.60
3 I feel listless	**0.77**	**0.84**		0.35		0.61
4 I have trouble starting things	**0.53**	**0.82**		0.37	0.43	0.79
5 I have trouble finishing things	**0.44**	**0.77**		0.33	0.50	0.79
6 I have energy		0.43	**0.91**	**0.88**		0.36
7 I am able to do my usual activities		0.27	**0.85**	**0.9**		0.36
8 I am frustrated by being too tired		0.62		0.38	**0.90**	**0.91**
9 I have to limit my social activity		0.61		0.40	**0.90**	**0.91**

The correlations between total score and each dimension were ≥ 0.64 and within dimensions ≥0.43 (p < 0.001 for all; Additional file 1: Table S2 ). There was a high correlation between the total score of the 13-item FACIT-F and the 9-item short version (r = 0.99; p < 0.001), indicating no loss of information after removing four items.

3) Rasch analysis

Initial inspection of the fit of data from the 9-item FACIT-F to the Rasch model showed a significant item–trait interaction with a total chi-square 308.2 with 81 degrees of freedom, p < 0.00001, suggesting some degree of misfit between the data and the model. This misfit to the model expectations may be due to items or respondents or both. The residual mean value for items was −0.517 with (a SD of 4.39, indicating inadequate fit to the model. The fit of the individual items was checked revealing misfit of several items to the model expectation. Most items showed fit residual values above ± 2.5 indicating significant deviation from the model. Rasch analysis was applied using an iterative process to achieve the best possible fit. Finally, the analysis of the pattern of residuals provided a reasonable explanation when the residuals loaded on mainly three subscales (components) with eigen value >1. This step showed significant improvement in fitting the Rasch model where items of each of the three components had residuals within the acceptable range of ±2.5. This finding was also supported by the *t*-test of local independence assumption further supporting that the FACIT-F is not a unidimensional construct in COPD. (Detailed description can be seen in Additional file 3).

The resulting residual mean value for patients was −0.49 with a SD of 1.36 indicating no significant misfit among the respondents in the sample. Individual person fit statistics showed that only 42 respondents had residuals outside the acceptable range. On removal of these persons, the chi squared interaction statistic did not change significantly with the PSI remaining high at 0.88 indicating the scale can constructively differentiate between groups. Further, when excluding patients with missing items (n = 24), the Cronbach α of the FACIT-F was 0.91.

None of the 9 items had disordered threshold indicating that response options were correctly ordered - the probability of endorsing more severe options increases in a logical progression. There was no sign of differential item functioning (DIF) indicating that the 9-item FACIT-F was not biased by the type of the responder (for example by the subjects’ gender).

For accuracy and comparative validity a backward-forward revising step was performed; i.e., the Rasch analysis was separately run on the 13-item FACIT-F which showed that two of the deleted items “I need to sleep during the day” and “I am too tired to eat” had disordered threshold and reordering their scoring system did not improve the fitness of the FACIT-F to the Rasch model. We also observed that the three deleted items “I need to sleep during the day”, “I am too tired to eat” and “I need help to do my usual activities” appeared unusual in the context of fatigue in COPD by having independently the strongest loading on three separate components (Detailed description can be seen in Additional file 4).

4) Reliability and Validity of the modified FACIT-F instrument

Reliability of FACIT-F scale:

Using Cronbach’s α for measuring internal consistency for the 9-item modified scale and each dimension we found values of 0.91, 0.91, 0.73 and 0.86 respectively at baseline, and values of 0.90, 0.87, 0.69 and 0.87 respectively at 3 years follow up. For the original 13-item scale, Cronbach’s α at baseline and at 3 years follow up were 0.92 and 0.93.

Validity of FACIT scale:

The original scale and the modified version and its dimensions showed good convergent validity achieving highly significant correlation with SGRQ and mMRC dyspnoea scale (Table 3).

**Table 3:** Correlations between total and dimensional scores of FACIT Fatigue Scale and SGRQ and mMRC dyspnoea scale

	**Total SGRQ score**	**SGRQ Symptom score**	**SGRQ Activity score**	**SGRQ Impact score**	**mMRC dyspnoea scale score**
Total fatigue score of 13-items FACIT-F scale)	- 0.7	- 0.45	- 0.58	- 0.7	- 0.48
Total fatigue score of 9-items modified FACIT-F	- 0.69	- 0.44	- 0.58	- 0.69	- 0.47
Modified FACIT-F: General dimension	- 0.65	- 0.44	- 0.52	- 0.65	- 0.44
Modified FACIT-F: Functional Ability dimension	- 0.42	- 0.25	- 0.38	- 0.41	- 0.31
Modified FACIT-F: Psychosocial dimension	- 0.63	- 0.37	- 0.53	- 0.62	- 0.44

All presented correlations have p <0.001.

mMRC dyspnoea scale: The modified Medical Research Council dyspnoea scale; SGRQ: St. George’s Respiratory Questionnaire.

Patients with high depression scores and exercise intolerance reported significantly more fatigue than patients with low depression scores and exercise tolerant patients (Table 4), as also demonstrated using the Receiver Operating Characteristic (ROC) curve (in the Additional file 1).

**Table 4:** FACIT-fatigue scores in COPD groups defined according to CES-D scores and 6MWD, mean values and standard deviations are shown

	**Depression**		**6MWD**	
	**Not depressed**	**Depressed**	**≥350 m**	**<350 m**
Total fatigue score of 13-items FACIT-F scale)	38.5 (8.9)	25.6 (9.6)	38.1 (9.5)	31.1 (10.8)
Total fatigue score of 9-items modified FACIT-F	25.5 (6.9)	15.6 (9.6)	25.3 (7.2)	20.1 (8.2)
Modified FACIT-F: General dimension	15 (4.2)	9.4 (4.6)	14.7 (4.4)	12 (5.1)
Modified FACIT-F: Functional Ability dimension	4.4 (1.9)	3.1 (1.6)	4.4 (1.9)	3.6 (1.8)
Modified FACIT-F: Psychosocial dimension	6.1 (2.2)	3.5 (2.4)	6.1 (2.2)	4.5 (2.6)

All presented values have a p value <0.001.

CES-D: the Centre for Epidemiologic Studies Depression scale; 6MWD: 6-Minute Walk Distance measured in meters.

## Discussion

This study presents a novel simple 9-item modified version of the FACIT-F scale to measure fatigue in COPD. The modified FACIT-F had a high level of convergent validity supported by significant correlations with widely used robust scales in COPD such as SGRQ and mMRC dyspnoea scale. It also had substantial discriminating validity between patients with depressive symptoms or limited exercise capacity versus patients who did not. In addition to the overall assessment of fatigue, the scale offers a novel assessment of multiple fatigue underlying components in COPD patients.

In this study, we explored the structure of the scale using a classic analysis framework followed by a PCA and Rasch analysis. Our analysis indicated that 4 items were likely suitable for deletion either due to lack of applicability or redundancy. Indeed, item “I feel tired” had a high correlation with 2 other items (items 1 and 5) particularly with item “I feel fatigued”; and the vast majority of our sample reported that certain items “I am too tired to eat” and “I need help to do my usual activities” were less likely to be relevant to fatigue in COPD. This loss of relevance may be due to a specific disease representation. For instance, fatigue can be significantly associated with certain symptoms such as loss of appetite in cancer [26] and neuromuscular dysfunction in multiple sclerosis [4]. Normal tiredness is usually resolved by sleep; however, pathogenic fatigue seems less responsive to sleep [16,27]. This may explain the low r^2^ coefficient, 0.29, of item “I need to sleep during the day” as patients with COPD commonly suffer from disturbed and poor quality of sleep [28].

There are a number of consistent statistical indicators of the validity of deleting the aforementioned items. First, the modified 9-item FACIT-F maintained the same level of correlation with the well-established robust scales validated in COPD including SGRQ and mMRC dyspnoea and was able to detect patients with severe COPD, high depressive symptoms or poor exercise performance. Secondly, a Cronbach’s α of > 0.7 indicates a good internal consistency. A shorter scale would be generally expected to have lower level of Cronbach’s α [24]; however, the 9-item modified FACIT-F maintained the high level of internal consistency of 0.91, similar to the original 13-item FACIT-F confirming the reliability of the scale after removal of the four items. Thirdly, PCA showed that all the 9 items loaded with no less than 0.4 on their components. This suggested that the retained items are able to capture descriptives of fatigue in COPD. Moreover, fatigue in COPD is likely a multi-dimensional phenomenon [16,29], and the dimensionality of the FACIT-F scale might reflect fundamental domains of fatigue in COPD.

The deletion of items was further supported by the Rasch analysis. This advanced technique showed that none of the items had disordered threshold or significant bias. Interestingly, the good person residual value suggested that the respondents were likely in a position of ease to understand and respond to the items. This was also observed by a high level of completeness where > 96% of the patients completed the questionnaire at both the visits without omitting items. The Rasch analysis also showed that the items in the original or modified FACIT-F did not form a unidimensional structure. This indicates that fatigue in COPD is more likely a multi-dimensional phenomenon [16], particularly in that both the modified FACIT-F and its components correlated well with robust scales such as SGRQ, mMRC dyspnoea and 6MWT. These underlying components seemed able to detect further features of fatigue in COPD. For instance, the psychosocial dimension of the modified FACIT-F was more sensitive (than the other dimensions) in differentiating between depressed and not depressed where the not depressed group scored almost double (indicating less fatigued) than the depressed as shown in Table 4. This suggests that the scale would offer an overall assessment of fatigue; and besides its underlying components could also provide further exploratory assessment for specific patterns of fatigue in COPD.

Other multi-dimensional scales have been used in assessing fatigue in COPD such as the 27-item Manchester COPD fatigue scale (MCFS) [16,30], the 20-item Multidimensional Fatigue Index (MFI-20) [31], the 41 visual analogue Piper Fatigue Scale (PFS) [32] and the 40 item Fatigue Impact Scale (FIS) [33]. None of these relatively long scales were developed as disease-specific scales except for the MCFS. The FACIT-F has shown significant correlation with MCFS (r = −0.81, p < 0.001), although the more comprehensive MCFS had shown better correlation with SGRQ, 6-MWD and BODE index [16]. However, generally, a shorter valid scale like the 9-item modified FACIT-F would have the advantages of ease of administration, would be less time consuming and probably have a better level of completeness, particularly when included in a battery of self-reported questionnaires administered to subjects at the same trial visit or when used in a busy clinic in daily practice.

Having a short questionnaire that retains excellent reliability for the assessment of fatigue has several advantages. In COPD, there has so far been a discrepancy between the few, and often non-validated, assessment questions used in the clinical management of COPD patients and the increasing number of questionnaires applied in observational studies as well as clinical trials. The exception has been the MRC dyspnoea scale [12] but this is a uni-dimensional scale capturing a single aspect of COPD – activity limitation. The recent work on the COPD Assessment Test (CAT) has resulted in a tool providing a standardised assessment of several respiratory symptoms likely to be used in both the clinical setting and in studies [34,35]. However, the CAT does not capture relevant aspects of fatigue. We suggest that the short fatigue scale resented in here will improve the assessment of this important aspect in COPD.

The limitations of our study include that this is a secondary analysis of an existing scale and the items of the COPD-version FACIT-F have not been generated by COPD patients. However, in a previous study FACIT-F had correlated well with a robust COPD specific fatigue scale (the 27 items Manchester COPD Fatigue Scale) [16], and here both original and modified FACIT-F demonstrated strong linear and binary correlation with well-established measures in COPD such as SGRQ, mMRC dyspnoea and 6MWT supporting its still validity in COPD field. Secondly, we did not report data on the test-retest repeatability and we did not measure the sensitivity of FACIT-F after a medical intervention such as pulmonary rehabilitation. However, the scale maintained the same excellent level of internal consistency at baseline and at 3 years follow up, and demonstrated good correlation with other robust scales. Therefore, we would expect the FACIT-F COPD-version to respond to interventions such as pulmonary rehabilitation, particularly since studies have recently indicated an improvement in fatigue following pulmonary rehabilitation [6]. However, more studies are required to study fatigue descriptors and influence and its response both totally and dimensionally after medical intervention using more sophisticated tools.

In conclusion, we found that both the original and the modified version of the FACIT-F were reliable and valid scales offering efficient measurement of fatigue in COPD. The modified version is shorter, offering total and dimensional fatigue assessment and is easier to complete in a busy daily practice and when included in a battery of scales in studies.

## Abbreviations

CAT: COPD Assessment Test; CESD: Centre for Epidemiologic Studies of Depression Scale; COPD: Chronic Obstructive Pulmonary Disease; DIF: Differential Item Functioning (DIF); ECLIPSE: Evaluation of COPD Longitudinally to Identify Predictive Surrogate Endpoints; FIS: Fatigue Impact Scale; FACIT-F: Functional Assessment of Chronic Illness Therapy-Fatigue scale; FEV_1_: Forced Expiratory Volume in One Second; FVC: Forced Vital Capacity; MCFS: Manchester COPD fatigue scale; 6MWD: 6 minute walk distance; mMRC: modified Medical Research Council Dyspnea scale; PFS: Piper Fatigue Scale; PCA: Principal Component Analysis; SGRQ-C: St. George’s Respiratory Questionnaire for COPD patients (SGRQ-C).

## Competing interests

Khaled Al-shair has no conflict of interest to declare in relation to this work. Hana Muellerova is an employee of GlaxoSmithKline R&D; she owns shares and stock option of GlaxoSmithKline. Janelle Yorke has no conflict of interest to declare in relation to this work. Stephen Rennard has served as a consultant or participated in advisory boards for: ABIM, Able Associates, Adelphi Research, Almirall, APT, Aradigm, Argenta, AstraZeneca, BI (ACCP), Biostrategies, BoomCom, Britnall and Nicolini, Capital Research, Chiesi, Clinical Advisors, CommonHealth, Complete Medical Group, Consult Complete, COPDForum, DataMonitor, Decision Resources, Defined Health, Dey, Dunn Group, Easton Associates, Enterprise Analysis, Equinox, Forest, Fulcrum, Gerson Lehman, GSK, Guidepoint, Hoffman LaRoche, IMS, Informed, Inspire, Insyght, KOL Connection, Leerink Swan, M. Pankove, MDRx Financial, MedaCorp, Medimmune, Mpex, Novartis, Nycomed, Oriel, Otsuka, Pearl, Pennside Partners, Pfizer, Pharma Ventures, Pharmaxis, Pick Research, Prescott, PwC, Propagate, Pulmatrix, Pulmonary Reviews, Quadrant, Reckner Associates, Recruiting Resource, Reviews and Trends in COPD/Convergent Health Solutions, Roche, Sacoor, Schering, Schlesinger Medical, Scimed, Smith Research, Sudler and Hennessey, Talecris, Theravance, UBC, Uptake Medical, Vantage Point. He has received lecture fees from: AAAAI, Am Col Osteopathic Physicians, Asan Medical Center, ATS, AstraZeneca, California Soc Allergy, Convergent Health Solutions for Reviews and Trends in COPD, COPD Foundation, Creative Educational Concepts, Dey, Duke, France Foundation, Information TV, University of California-Los Angeles, Network for Continuing Education, Novartis, Nycomed, Otsuka, Pfizer, Sarasota Mem Hospital, Spanish Thoracic Society, University of Washington, University of Alabama-Birmingham, University of Pittsburgh, University of British Columbia, University of California-Davis, VA Sioux Falls. He has received industry-sponsored grants from: AstraZeneca, Biomarck, Centocor, GlaxoSmithKline, Mpex, Nabi, Novartis, Otsuka, Pfizer. Nicola A. Hanania has received research grant support as well as served as a consultant and on the speaker bureau for GSK. Emiel Wouters has recevied honoraria for presenting and consulting from AstraZeneca, Boehringer-Ingelheim, GlaxoSmithKline, Novartis, Nycomed and Pfizer and his department has received research grants from GlaxoSmithKline. Amir Sharafkhaneh is on advisory board of GSK and Dey. Jørgen Vestbo has recevied honoraria for presenting and consulting from AstraZeneca, Boehringer-Ingelheim, Bioxydyn, Chiesi, GlaxoSmithKline, Novartis, Nycomed and Pfizer and his department has received research grants from GlaxoSmithKline; his wife has previously worked for several pharmaceutical companies, including GlaxoSmithKline.

## Authors’ contributions

KA participated in the study design and data collection, and performed the statistical analyses and wrote the manuscript. HM, JY, SR, EW, NH, AS and JV participated in study design, data analysis and the manuscript writing, editing and reviewing. All authors read and approved the manuscript.

## Funding

The ECLIPSE study was funded by GlaxoSmithKline (registered on ClinicalTrials.gov with identifier NCT00292552; GlaxoSmithKline study code SCO104960).

## Supplementary Material

Additional file 1 E-supplement. Click here for file

Additional file 2 **Appendix 1.** PCA and Reliability and validity of FACIT scale of 13-item FACIT-F. Click here for file

Additional file 3 **Appendix 2.** Additional description on Rasch analysis of the 9-item FACIT-F scale. Click here for file

Additional file 4 **Appendix 3.** Additional description on Rasch analysis of the 13-item FACIT-F scale. Click here for file
