# Supplementary material for: Examining fatigue in COPD: development, validity and reliability of a modified version of FACIT-F scale
Source: Health Qual Life Outcomes. 2012 Aug 23;10:100. doi: 10.1186/1477-7525-10-100 (PMC3491053; doi:10.1186/1477-7525-10-100)
Supplement: Additional file 1 — E-supplement. [file 1477-7525-10-100-S1.doc]

**Examining fatigue in COPD: development, validity and reliability of a modified version of FACIT-F scale**

Khaled Al-shair, Hana Muellerova, Janelle Yorke, Stephan Rennard, Emiel Wouters, Nicola A. Hanania, Amir Sharafkhaneh, Jorgen Vestbo, ECLIPSE investigators

**E-supplement:**

**Methods:**

**Additional statistical analyses**

Spearman correlation coefficient was used to examine the correlation between the total FACIT-F and total and dimensional modified FACIT-F with mMRC dyspnoea scores. Paired t-test was used to compare the mean scores of the FACIT-F, modified FACIT-F and its dimensions, as well as SGRQ and CES-D at both visits. The association of total FACIT- F and total and dimensional modified FACIT-F with COPD severity (using GOLD stages 2,3,4) was examined using the analysis of variance (ANOVA). We used the independent sample t-test to compare the mean fatigue score in groups defined according to CES-D depression scores and 6MWD. SPSS version 15 (SPSS Inc, USA) was used.

**Results:**

**Table 1.** Summary of the traditional psychometric properties assessment

| Item number | Item | Not applicable (n, %) | Redundancy  (High correlation with other item(s). (r ≥ 0.7 is presented) | r^2^ coefficient | Floor or ceiling effect |
| --- | --- | --- | --- | --- | --- |
| 1 | I feel fatigued | 137 (6.5%) | 0.74 with item (4) | 0.59 | 2.28 (1.13) |
| 2 | I feel weak all over | 739 (35.3%) |  | 0.59 | 2.82 (1.27) |
| 3 | I feel listless | 904 (43.3%) |  | 0.58 | 2.98 (1.13) |
| 4 | I feel tired | 143 (6.9%) | 0.72 with item (5) | 0.65 | 2.34 (1.16) |
| 5 | I have trouble starting thing because I am tired | 734 (35.1%) | 0.78 with item (6) | 0.71 | 2.75 (1.2) |
| 6 | I have trouble finishing thing because I am tired | 722 (34.7%) |  | 0.64 | 2.74 (1.2) |
| 7 | I have energy | 207 (9.9%) |  | 0.35 | 1.86 (1) |
| 8 | I am able to do my usual activities | 180 (8.6%) |  | 0.29 | 2.24 (1.14) |
| 9 | I need to sleep during the day | *687*  *(32.6%)* |  | 0.29 | *2.77 (1.14)* |
| 10 | I am too tired to eat | 1618 (76.8%) |  | 0.28 | 3.66 (0.72) |
| 11 | I need help to do my usual activities | 1290 (61.5%) |  | 0.4 | 3.34 (1) |
| 12 | I am frustrated by being too tired to do the things I want to do | 817 (38.8%) | 0.76 with item (13) | 0.68 | 2.65 (1.38) |
| 13 | I have to limit my social activity because I am tired | 885 (42%) |  | 0.6 | 2.79 (1.29) |

**Table 2.** Correlation between the components in the three factors solution for the 9-item FACIT-F Scale.

|  | **General dimension** | **Functional Ability dimension** | **Psychosocial dimension** |
| --- | --- | --- | --- |
| **General dimension** | 1 | 0.43 | 0.75 |
| **Functional Ability dimension** | 0.43 | 1 | 0.43 |
| **Psychosocial dimension** | 0.75 | 0.43 | 1 |
| **Total FACIT score** | 0.96 | 0.64 | 0.87 |

All presented correlations have a p value <0.001

**ROC curve analysis of 13-item FACIT-F and modified 9-item FACIT-F and its domains:**

*To investigate this subject we have chosen two well-established measures that have been widely used in COPD studies and have a strong correlation with fatigue; one is subjective (depression using CES-D) and the other is objective (6MWD).*

*It has been shown that ≥ 16 score indicates the presence of depressive symptoms (1). Using the value (<16) as the (Value of State Variable), the ROC curve analysis indicated a very good ability of modified FACIT-F to detect the difference in mood status between patients who had less depressive symptoms and who had more depressive symptoms (Table 3, Figure 1). The area under the curve (AUC) of total modified FACIT-F score showed very good discriminatory ability (AUC = 0.833; range 0.81 – 0.85, p<0.001).*

***Table 3****, ROC curve analysis of 13-item FACIT-F and modified 9-item FACIT-F and its domains; discrimination between depressed and not depressed COPD patients.*

|  | *AUC* | *CI* | *P* |
| --- | --- | --- | --- |
| *13-item FACIT-F* | *0.832* | *0.81 – 0.85* | *<0.001* |
| *Modified 9-item FACIT-F* | *0.833* | *0.81 – 0.85* | *<0.001* |
| *Modified FACIT-F: General dimension* | *0.82* | *0.8 – 0.84* | *<0.001* |
| *Modified FACIT-F: Functional Ability dimension* | *0.7* | *0.68 – 0.73* | *<0.001* |
| *Modified FACIT-F: Psychosocial dimension* | *0.79* | *0.77 – 0.81* | *<0.001* |

***Figure 1,*** *ROC curve analysis of 13-item FACIT-F and modified 9-item FACIT-F and its domains; discrimination between depressed and not depressed COPD patients.*

*
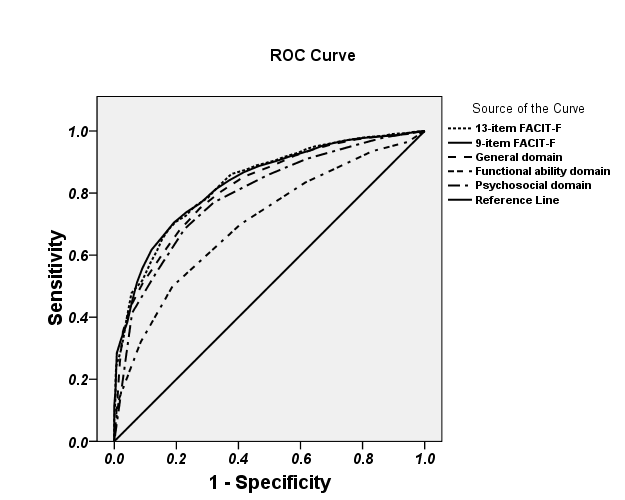
*

*It has been shown that ≥ 350 meter in the 6MWT indicates good exercise capacity in COPD (2). Using the value (≥ 350 meter) as the (Value of State Variable), the ROC curve analysis indicated moderately a good ability of modified FACIT-F to detect the difference in exercise capacity between patients who walked ≥ 350 meter in the 6MWT and who walked <350m (Table 4, Figure 2). The area under the curve (AUC) of total modified FACIT-F score showed moderately good discriminatory capacity (AUC = 0.68; range 0.66 – 0.7, p<0.001).*

***Table 4****, ROC curve analysis of 13-item FACIT-F and modified 9-item FACIT-F and its domains; discrimination between patients walked ≥ 350m and who walked<350m in 6MWT.*

|  | *AUC* | *CI* | *P* |
| --- | --- | --- | --- |
| *13-item FACIT-F* | *0.69* | *0.66 – 0.71* | *<0.001* |
| *Modified 9-item FACIT-F* | *0.68* | *0.66 – 0.7* | *<0.001* |
| *Modified FACIT-F: General dimension* | *0.66* | *0.64 – 0.69* | *<0.001* |
| *Modified FACIT-F: Functional Ability dimension* | *0.62* | *0.6 – 0.65* | *<0.001* |
| *Modified FACIT-F: Psychosocial dimension* | *0.67* | *0.65 – 0.7* | *<0.001* |

***Figure 2,*** *ROC curve analysis of 13-item FACIT-F and modified 9-item FACIT-F and its domains; discrimination between COPD patients walked ≥ 350m and who walked<350m in 6MWT.*

*
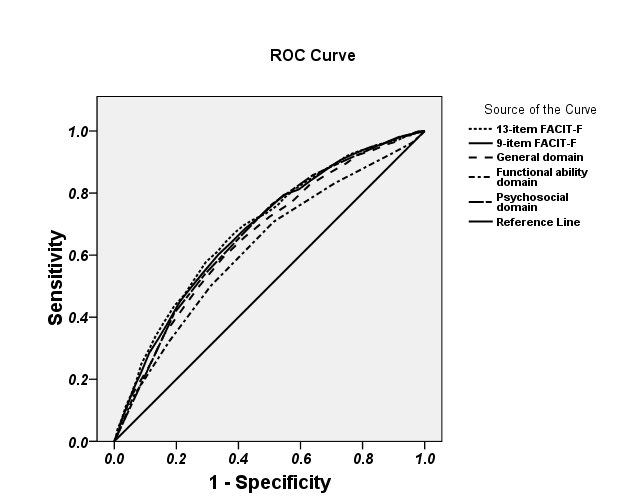
*

**References**
