# Supplementary material for: Examining fatigue in COPD: development, validity and reliability of a modified version of FACIT-F scale
Source: Health Qual Life Outcomes. 2012 Aug 23;10:100. doi: 10.1186/1477-7525-10-100 (PMC3491053; doi:10.1186/1477-7525-10-100)
Supplement: Additional file 2 — Appendix 1. PCA and Reliability and validity of FACIT scale of 13-item FACIT-F. [file 1477-7525-10-100-S2.doc]

**Examining fatigue in COPD: development, validity and reliability of a modified version of FACIT-F scale**

Khaled Al-shair, Hana Muellerova, Janelle Yorke, Stephen I. Rennard, Emiel F.M. Wouters, Nicola A. Hanania, Amir Sharafkhaneh, Jørgen Vestbo, for the ECLIPSE investigators

**Appendix 1: PCA and Reliability and validity of FACIT scale of 13-item FACIT-F**

**Results:**

The 13 items Functional Assessment of Chronic Illness Therapy (FACIT)-Fatigue scale was introduced to 2107 patient with clinically stable COPD. The items of the scale were subjected to the principal components analysis (PCA) using SPSS version 15.

Prior to performing PCA the suitability of data for factor analysis was assessed. Inspection of the correlation matrix revealed the presence of many coefficient correlation of 0.3 and above. The Kaiser-Meyer-Oklin value was 0.93v, exceeding the recommended value of 0.6 (Kaiser 1970, 1974) and Bartlett’s Test of Sphericity (Bartlett, 1954) reached statistical significance (<0.001), supporting the factorability of the correlation matrix.

PCA revealed the presence of two components with eigenvalues exceeding 1. As inspection of the scree plot revealed a clear break after the second component. Using Catell’s (1966) scree test, it was decided to retain two components for further investigation. To aid on the interpretation of these components, several rotation techniques such as Varimax and Direct Oblimin were performed. The rotated solution revealed the presence of simple structure with two components showing a number of strong loadings and most of the items loading substantially on component 1. The interpretation of the two components solution consistently showed that negative effect (impact) items loading strongly on component 1 and positive effect items loading on component 2. This analysis may also support the use of the negative effect items and positive effect items as separate scales.

**Table 1.** Pattern and Structure Matrix for PCA with Oblimin Rotation of two factors solution of the 13-items FACIT-F scale (loading values ≤ 0.3 were not demonstrated).

|  | Items | **Components** | | | |
| --- | --- | --- | --- | --- | --- |
|  |  | **1** | | **2** | |
|  |  | **Pattern** | **Structure** | **Pattern** | **Structure** |
| 1 | I feel tired | **0.880** | **0.85** |  |  |
| 2 | I have trouble starting things | **0.852** | **0.87** |  | 0.37 |
| 3 | I feel fatigued | **0.829** | **0.81** |  |  |
| 4 | I have trouble finishing things | **0.821** | **0.83** |  | 0.35 |
| 5 | I feel weak all over | **0.810** | **0.81** |  | 0.31 |
| 6 | I feel listless | **0.796** | **0.80** |  | 0.32 |
| 7 | I am frustrated by being too tired | **0.754** | **0.81** |  | 0.43 |
| 8 | I have to limit my social activity | **0.722** | **0.79** |  | 0.46 |
| 9 | I need sleep during the day | **0.641** | **0.58** |  |  |
| 10 | I am too tired to eat | **0.545** | **0.57** |  |  |
| 11 | I need help doing usual activities | **0.523** | **0.62** |  | 0.47 |
| 12 | I am able to do my usual activities |  | 0.33 | **0.910** | **0.898** |
| 13 | I have energy |  | 0.43 | **0.793** | **0.837** |

**Table 2.** Correlation between the components in the two factors solution for the 13-items FACIT Scale (COPD patients at baseline).

|  | **Component 1** | **Component 2** |
| --- | --- | --- |
| **Component 1** | 1 | 0.59 |
| **Component 2** | 0.59 | 1 |
| **Total FACIT score** | 0.99 | 0.59 |

All presented correlations have a p value <0.001

Moreover, no difference was seen when the PCA was separately applied on GOLD stages (2,3,4; n= 925, 894, 292 patients) respectively i.e., the items loaded on the 2 components with almost similar variance definition.

The PCA was also applied on data collected from 1636 COPD patients at 3 years follow up visit. The analysis showed consist results with baseline. Similar loadings were seen for either combined COPD patients (at baseline and follow up) or controls.
