# Supplementary material for: Examining fatigue in COPD: development, validity and reliability of a modified version of FACIT-F scale
Source: Health Qual Life Outcomes. 2012 Aug 23;10:100. doi: 10.1186/1477-7525-10-100 (PMC3491053; doi:10.1186/1477-7525-10-100)
Supplement: Additional file 3 — Appendix 2. Additional description on Rasch analysis of the 9-item FACIT-F scale. [file 1477-7525-10-100-S3.doc]

**Examining fatigue in COPD: development, validity and reliability of a modified version of FACIT-F scale**

Khaled Al-shair, Hana Muellerova, Janelle Yorke, Stephen I. Rennard, Emiel F.M. Wouters, Nicola A. Hanania, Amir Sharafkhaneh, Jørgen Vestbo, for the ECLIPSE investigators

**Appendix 2: Additional description on Rasch analysis of the 9-item FACIT-F scale:**

**Methods:**

The FACIT-F was administered to 2108 COPD patients at baseline. Using random sampling (with consideration of GOLD stages, gender and age categories), we divided the sample to 4 groups. We investigated the scale in the 1^st^ group and validated the results in the other groups (each group had at least 500 COPD patients).

Rasch analysis was conducted using RUMM2030 software to assess the overall fit of the model, the response scale, individual item fit, thresholds, differential item functioning (DIF), local independence and person separation.

This analysis was also conducted for the whole group and no significant difference was observed.

This work has in principle followed the introduction to Rasch analysis by Pallant and Tennant (Pallant and Tennant, 2007) and others work (Conaghan et al., 2007, Mills et al., 2010, Yorke et al., 2011).

**The results:**

**Overall fit of the 13-item FACIT-F scale:**

Initial inspection of the fit of the data from all 9 items to the Rasch model shows a

significant item–trait interaction with a total chi-square (308.2 (df 81) with p = 0.00000), suggesting that there is some degree of misfit between the data and the model. This could be caused by misfit to model expectations of items or respondents or both. **The residual mean value** for items was -0.517 with a (SD of 4.39, much higher than the expected value of 1, given inadequate fit to the model). This deviation is supported by a significant chi squared interaction of (308.2 (df 81) with p = 0.00000). **The residual mean value for persons** was -0.49 with a SD of 1.36 indicating no serious misfit among the respondents in the sample.

**The Person-Separation index** was 0.88 (approximately equal when including or excluding the extremist values) indicating the scale can constructively able to differentiate between groups. When excluding persons with missing items (n=24) we left with a sample of (519 patients) in which Cronbach alpha of the FACIT was 0.907 (when including the extreme values, it was 0.911), and the person-Separation index was 0.882 (when including the extreme value it was 0.885).

**Thresholds:**

Initially, the pattern of thresholds is examined to see if disordering may be affecting fit. We found that none of the 9 items had disordered threshold as shown in figure 1.

**Figure1, Threshold ordering,**

**
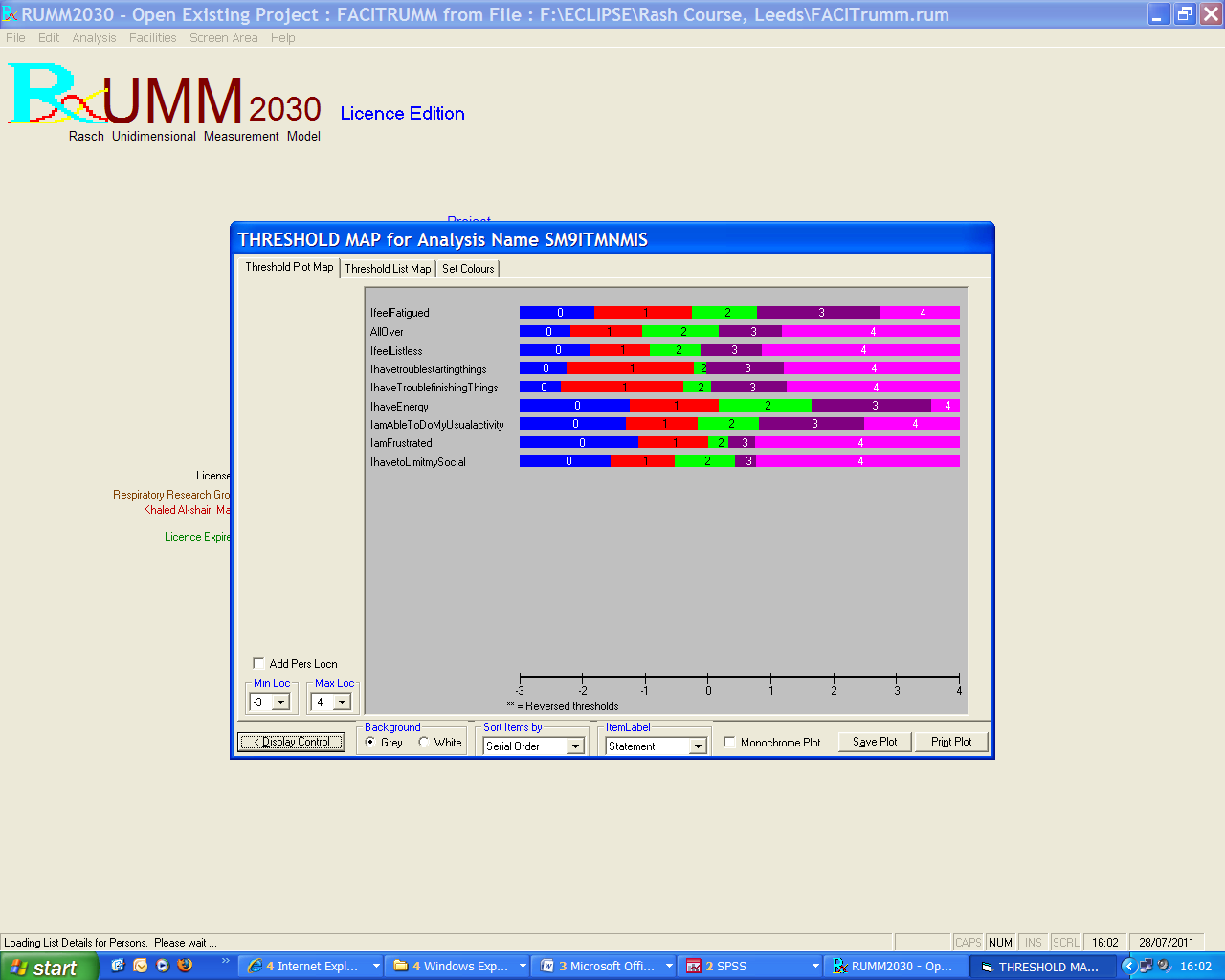
**

The ordering of thresholds is graphically demonstrated in the category probability

curves. For instance, figure 2 shows how item thresholds for item (I feel weak all over) are properly ordered, where each response category (0,1,2,3,4) systematically has a point along the ability continuum where it is the most likely response, as indicated by a peak in the curve.

**Figure 2,** Category probability curve for item 2 (I feel weak all over).


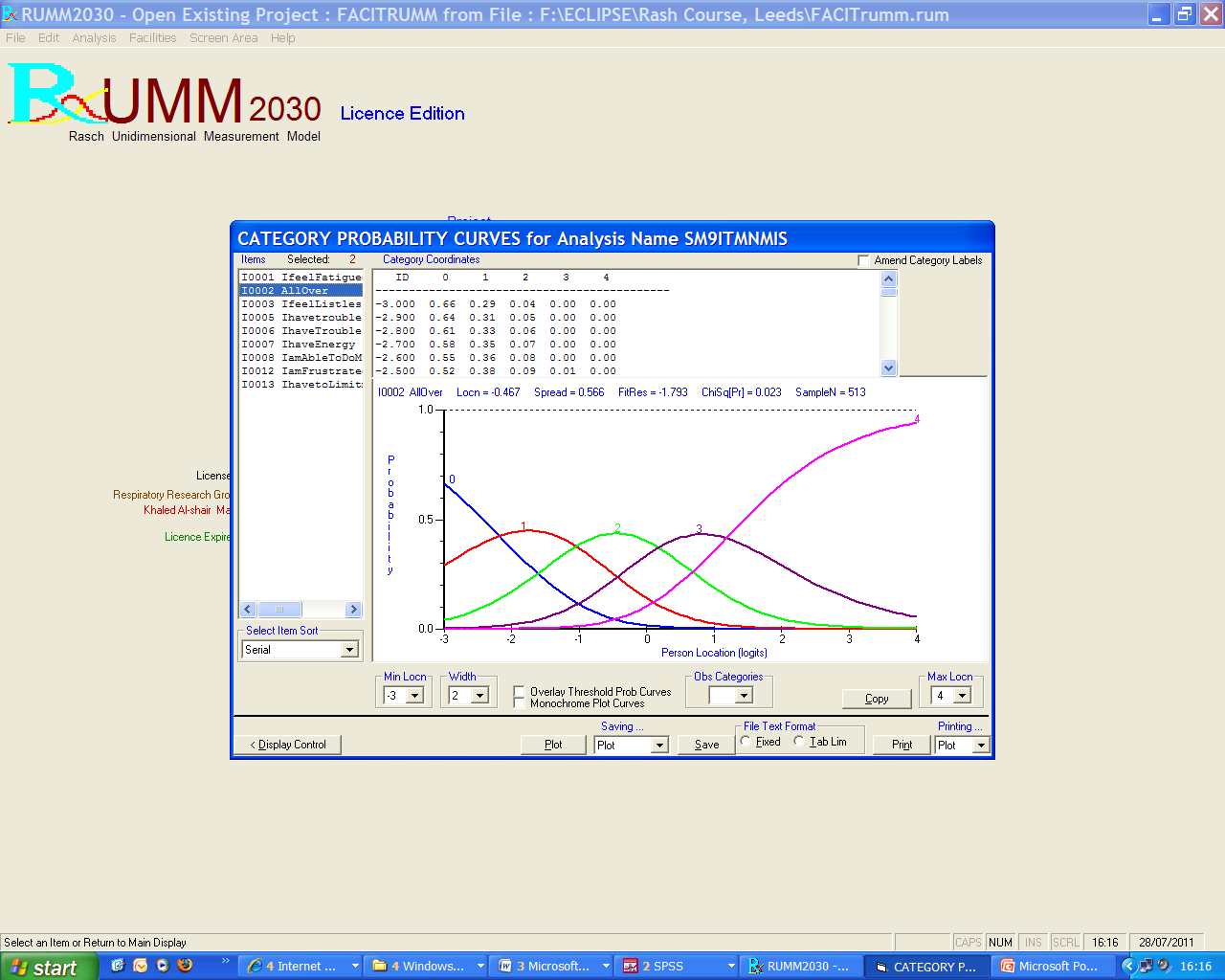
In

**Individual item fit**

The fit of the individual items was checked revealing that several items misfit to model expectation as shown in table 1,

**Table 1, Fit of the FACIT-F items to the Rasch model
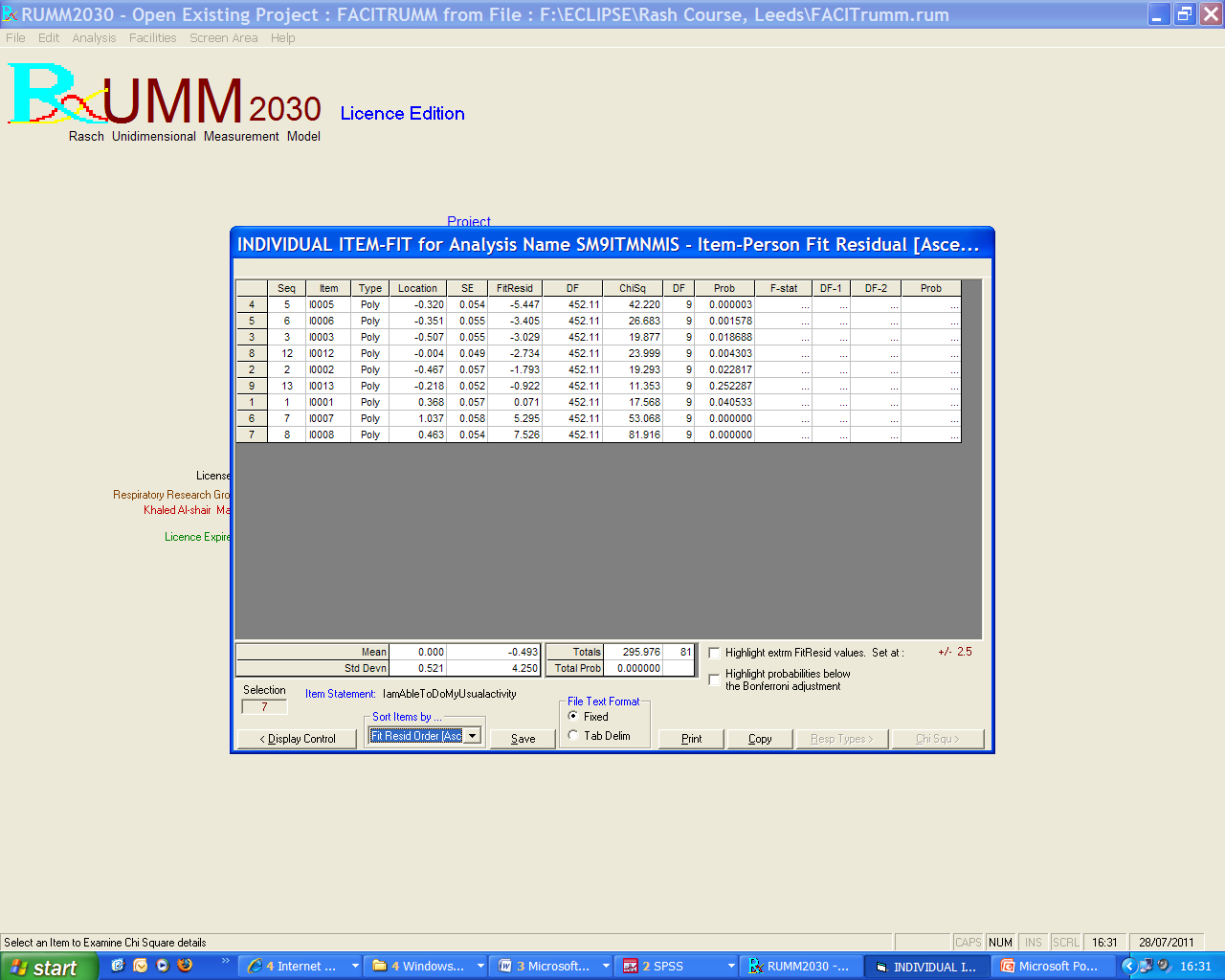
**

Most of the items showed fit residual values above ± 2.5. The positive fit residual values obtained for items (7, 8) suggest low levels of discrimination. The plot of observed group responses deviates from the model curve, and this observed response is flatter than the ICC, showing under-discrimination (Figures 5, 6) in the supplement). Items 2, 3, 5, 6 showed marginal over-discrimination as illustrated in (figures 8, 9, 11, 12 in the supplement).

**Person fit:**

Individual person fit statistics showed that 42 respondents had residuals outside the

acceptable range. On removal of these persons, the chi squared interaction statistic

did not significantly improve (277, df (81) p value = 0.0000); with the PSI remaining high at 0.88 as shown in figure 14a in the supplement.

**Test of local independence assumption,**

Analysis of the pattern of residuals showed that the residuals loaded

on mainly three subscales (components) as sown in figure 3 and 4,

Items of these three subsets were then separately fitted to the required residual value ±2.5 and chi square p value for each dimension was dramatically improved (0.06, 0.001 and 0.15 respectively, with items in each dimension had Bonferoni adjusted p value > 0.01).

The differences in person estimates derived from these analyses were significant, where the number of significant t-tests between e.g., the 1st subset and 2^nd^ subset was more than 5% and stood at 13.97% supporting a that the FACIT-F is not a unidimensional construct.

**Figure 3, Local independence test,**

**
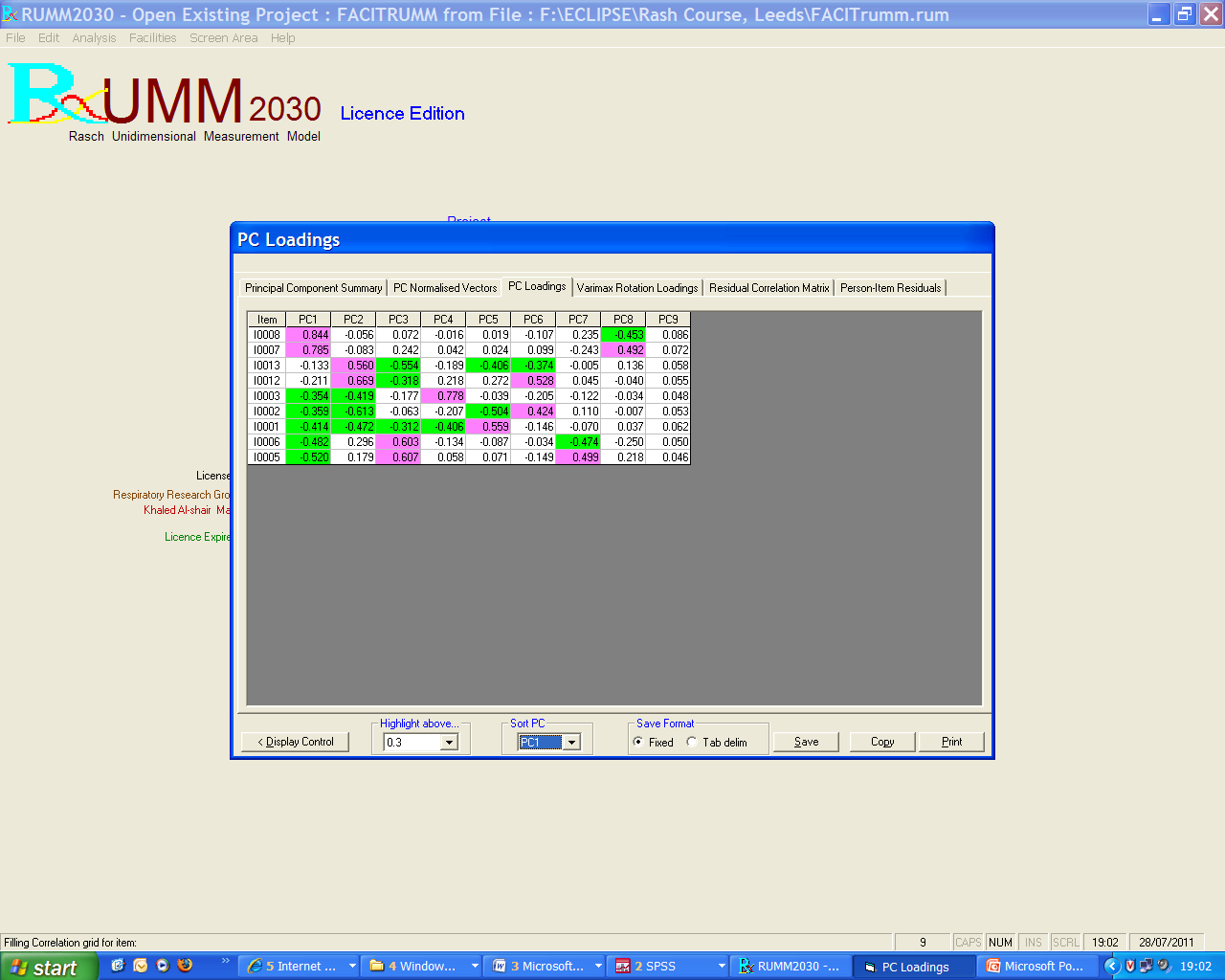
**

**Figure 4, components of the FACIT-F scale**


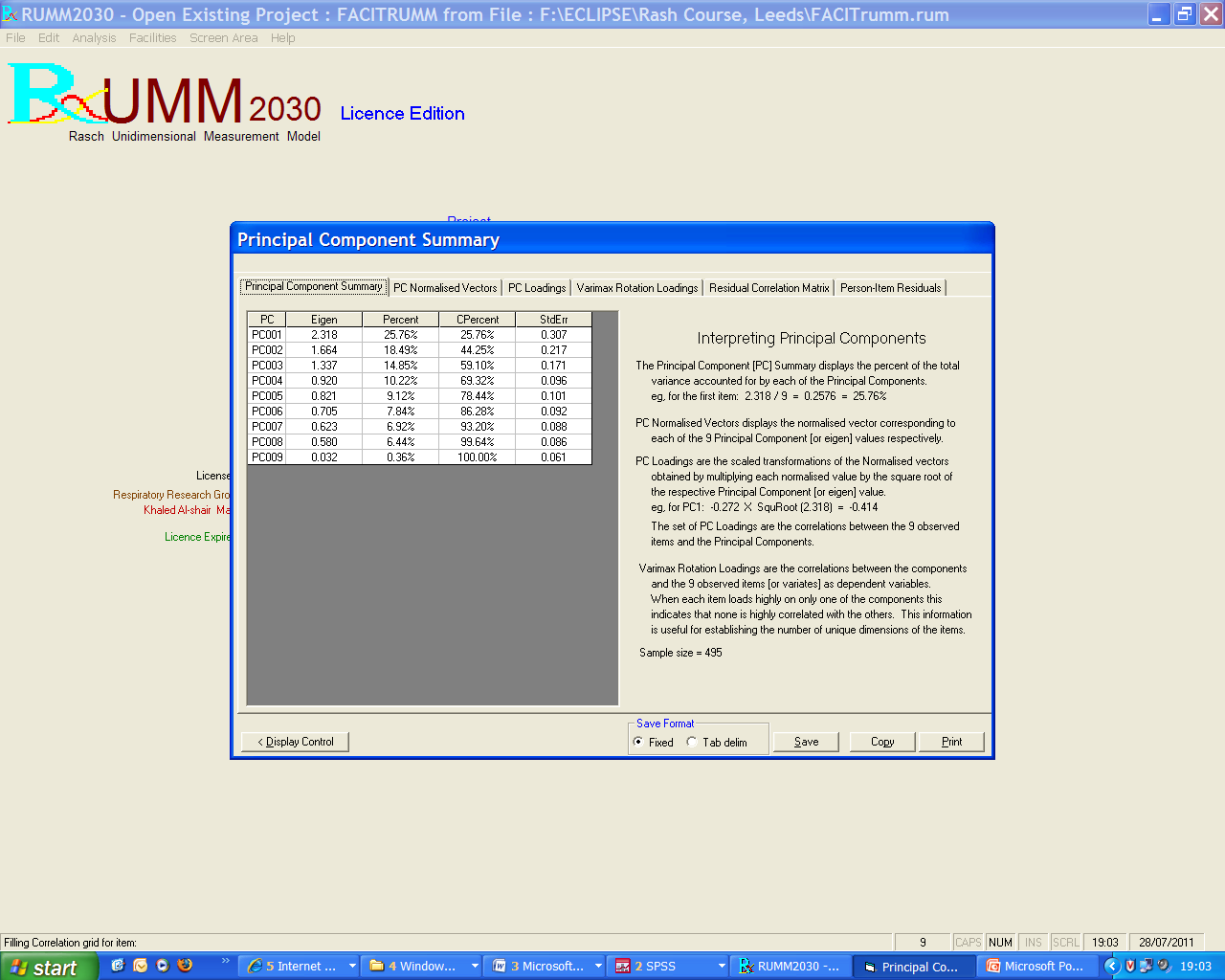


**References:**

CONAGHAN, P. G., EMERTON, M. & TENNANT, A. (2007) Internal construct validity of the Oxford Knee Scale: evidence from Rasch measurement. *Arthritis Rheum,* 57**,** 1363-7.

MILLS, R. J., YOUNG, C. A., PALLANT, J. F. & TENNANT, A. (2010) Rasch analysis of the Modified Fatigue Impact Scale (MFIS) in multiple sclerosis. *J Neurol Neurosurg Psychiatry,* 81**,** 1049-51.

PALLANT, J. F. & TENNANT, A. (2007) An introduction to the Rasch measurement model: an example using the Hospital Anxiety and Depression Scale (HADS). *Br J Clin Psychol,* 46**,** 1-18.

YORKE, J., JONES, P. W. & SWIGRIS, J. J. (2011) Development and validity testing of an IPF-specific version of the St George's Respiratory Questionnaire. *Thorax,* 65**,** 921-6.
