# Supplementary material for: Examining fatigue in COPD: development, validity and reliability of a modified version of FACIT-F scale
Source: Health Qual Life Outcomes. 2012 Aug 23;10:100. doi: 10.1186/1477-7525-10-100 (PMC3491053; doi:10.1186/1477-7525-10-100)
Supplement: Additional file 4 — Appendix 3. Additional description on Rasch analysis of the 13-item FACIT-F scale. [file 1477-7525-10-100-S4.doc]

**Examining fatigue in COPD: development, validity and reliability of a modified version of FACIT-F scale**

Khaled Al-shair, Hana Muellerova, Janelle Yorke, Stephen I. Rennard, Emiel F.M. Wouters, Nicola A. Hanania, Amir Sharafkhaneh, Jørgen Vestbo, for the ECLIPSE investigators

**Appendix 3: Additional description on Rasch analysis of the 13-item FACIT-F scale**

**Methods:**

The FACIT-F was administered to 2107 COPD patients at baseline. Using random sampling (with consideration of GOLD stages, gender and age categories), we divided the sample to 4 groups. We investigated the scale in the 1^st^ group and validated the results in the other groups (each group had at least 500 COPD patients).

Rasch analysis was conducted using RUMM2030 to assess the overall fit of the model, the response scale, individual item fit, thresholds, differential item functioning (DIF), local independence and person separation.

This work has in principle followed the introduction to Rasch analysis by Pallant and Tennant (Pallant and Tennant, 2007) and others work (Conaghan et al., 2007, Mills et al., 2010, Yorke et al., 2011).

**The results:**

**Overall fit of the 13-items FACIT-F scale:**

Initial inspection of the fit of the data from all 13 items to the Rasch model shows a

significant item–trait interaction with a total chi-square (453.78 (df 117) with p = 0.00000), suggesting that there is some degree of misfit between the data and the model. This could be caused by misfit to model expectations of items or respondents or both. The residual mean value for items was -0.629 with a (SD of 4.39, much higher than the expected value of 1. This deviation is supported by a significant chi squared interaction of 453.78 (df 117) with p = 0.00000. The residual mean value for persons was -0.397 with a SD of 1.296 indicating no serious misfit among the respondents in the sample.

The Person-Separation index was 0.903 (approximately equal when including or excluding the extremist values) indicating the scale can constructively able to differentiate between groups. When excluding persons with missing items (n=21) we left with a sample of (484 patients) in which Cronbach alpha of the FACIT was 0.915 (when including the extreme values, it was 0.913), and the person-Separation index was 0.893 (when including the extreme value it was 0.891).

**Thresholds:**

Initially, the pattern of thresholds is examined to see if disordering may be affecting fit. We found that two items were having disordered threshold (items: **I am too tired to eat; I need help doing usual activities) as shown in figure 1.**

**Figure1, Threshold ordering,**

**
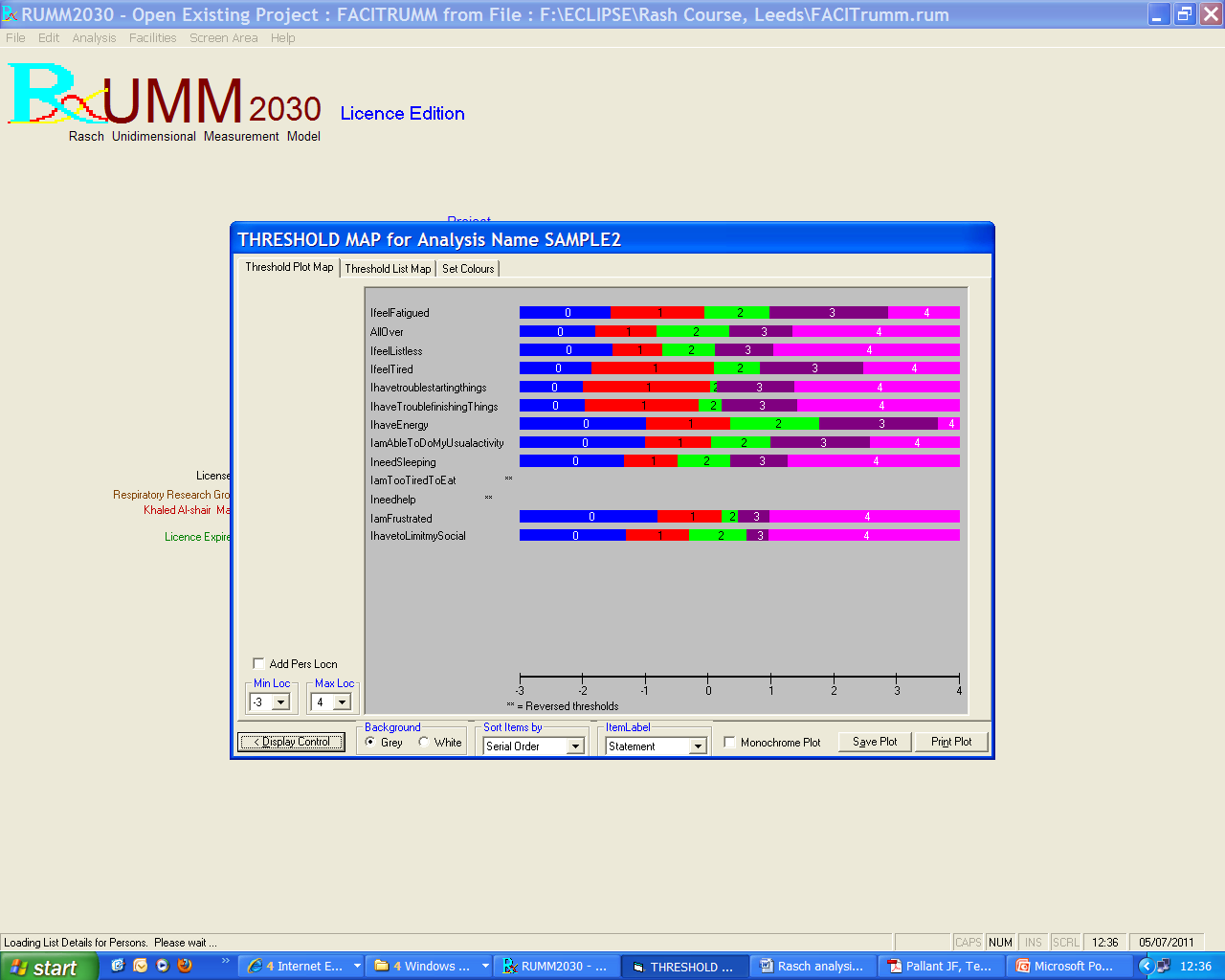
**

The ordering of thresholds is graphically demonstrated in the category probability

curves shown in Figs 1 and 3 in the supplement. Figure 1 (in supplement) shows clearly how item thresholds for item (I feel fatigued) are properly ordered, where each response category (0,1,2,3,4) systematically has a point along the ability continuum where it is the most likely response, as indicated by a peak in the curve. In contrast, Figure. 2, and 3 (in supplement) for items (I am too tired to eat) and (I need help to do my usual activities) showed disordered thresholds.

The point at which the lines for adjacent response categories cross in Fig1 (supplement) indicates that the transition between categories 2 and 3 is lower on the trait (more fatigue) than between categories 1 and 2, which is not how the variable is intended to work.

Consequently, scores for this item were recoded by collapsing the responses to the

third (scored 1) and fourth and fifth (scored 2) response category to form three, rather than five categories (coded 01122) as shown in figure 2 and (in figures 4a, and 4b in the supplement). However, no improvement was seen in the overall model fit where overall items residual mean value (SD) was -0.663 (4.57) and (p = 0.00000) and the PSI remained at 0.90. Alternative recoding procedures were also checked; however, no other solution improved the overall fit of the model.

**Figure 2, Threshold order after recoding items (eating, need help):**

**
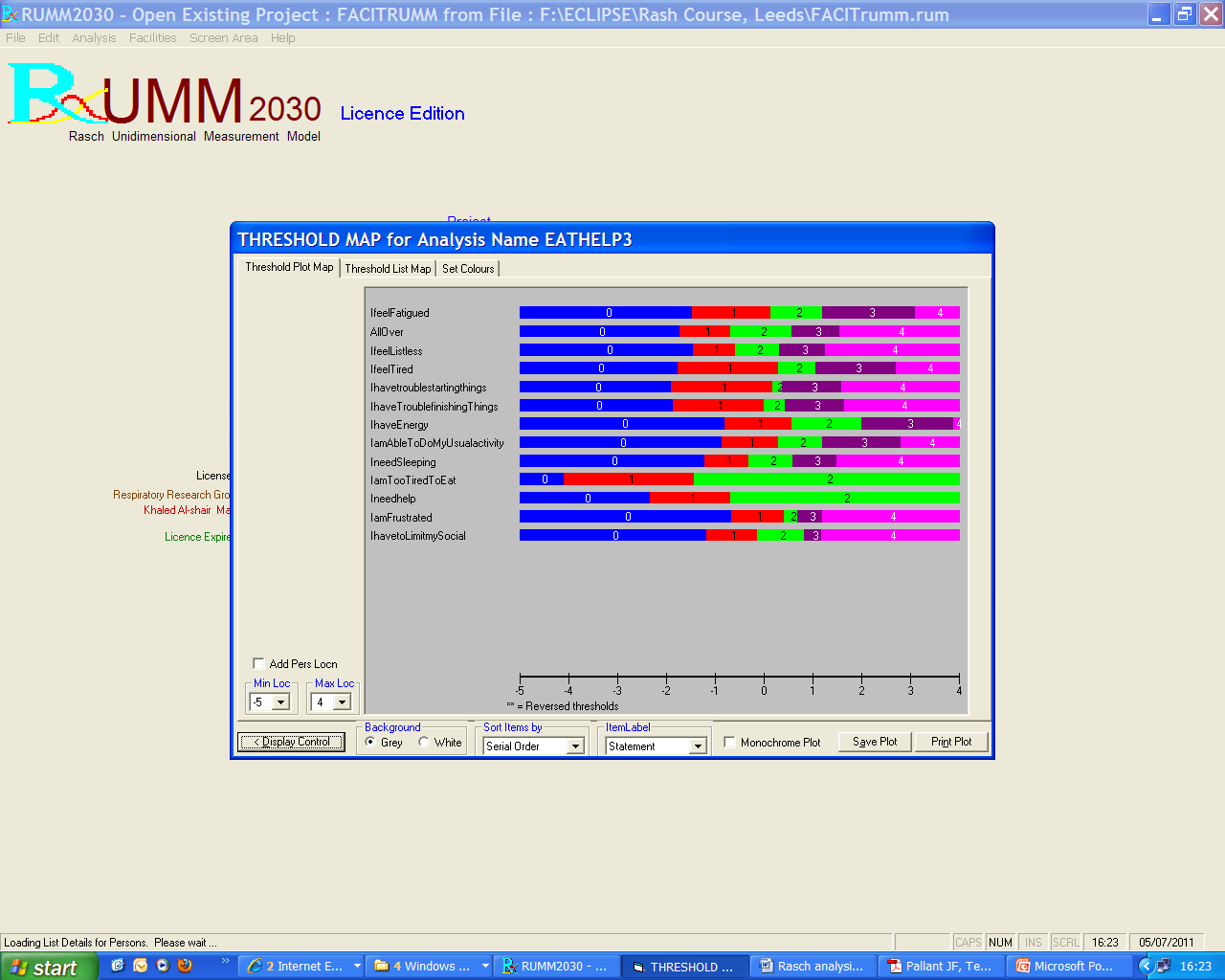
**

**Individual item fit**

Following recoding of items (10 and 11), the fit of the individual items was checked revealing that several items still misfit to model expectation (Table 1) (Original scoring individual item fit is shown in the supplement (table 1)).

**Table 1, Fit of the FACIT-F items to the Rasch model after rescoring of items 10 and 11**


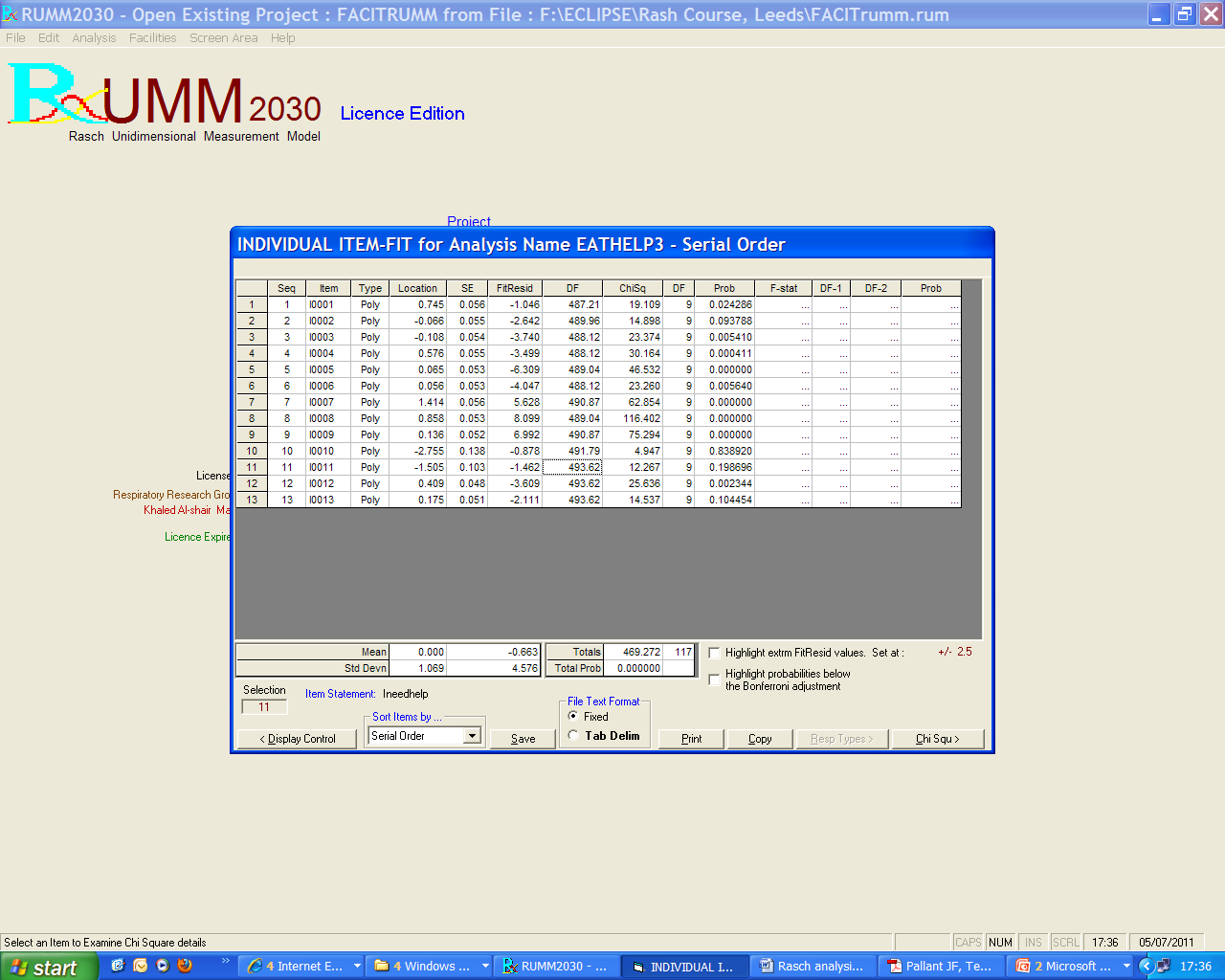


Most of the items showed fit residual values above ± 2.5, and the probability value for items 4.5.7,8,9 is less than the Bonferroni adjusted value of .004, indicating significant deviation from the model.

The positive fit residual values obtained for items (7, 8 and 9) suggest low levels of

discrimination. The plot of observed group responses deviates from the model curve, and this observed response is flatter than the ICC, showing under-discrimination (Figures 5,6.7) in the supplement). Thus responses from the lowest group (low scores of FACIT) are above what is expected by the model and those for the highest group (high scores of FACIT), are below model expectation. Items 2, 3, 4, 5 and 12 showed marginal over-discrimination as illustrated in (figures 8, 9, 10, 11, 12, 13 in the supplement).

**Person fit:**

Individual person fit statistics showed that 39 respondents had residuals outside the

acceptable range. On removal of these persons, the chi squared interaction statistic

did not improve (447, df (117) p= 0.0000); with the PSI remaining high at 0.90 as shown in figure 14b in the supplement. Moreover, at the individual item level, the previous misfit items did not significantly improve. As persons removal; however, did not improve the overall fit of the scale, so it was decided to retain the items.

**Test of local independence assumption,**

Analysis of the pattern of residuals showed that the residuals loaded in opposite

directions on mainly two subscales (components) as sown in figure 3 and 4,

These two subsets of items (defined by positive and negative loadings on

the first residual component) were then separately fitted to the Rasch model.

The differences in person estimates derived from these analyses were significant, where the number of significant t-tests between the 1st subset and 2^nd^ subset was more than 5% and stood at 13.2% supporting a that the FACIT is not a unidimensional construct as shown in figure 15 (in the supplement).

**Figure 3, Local independence test,**

**
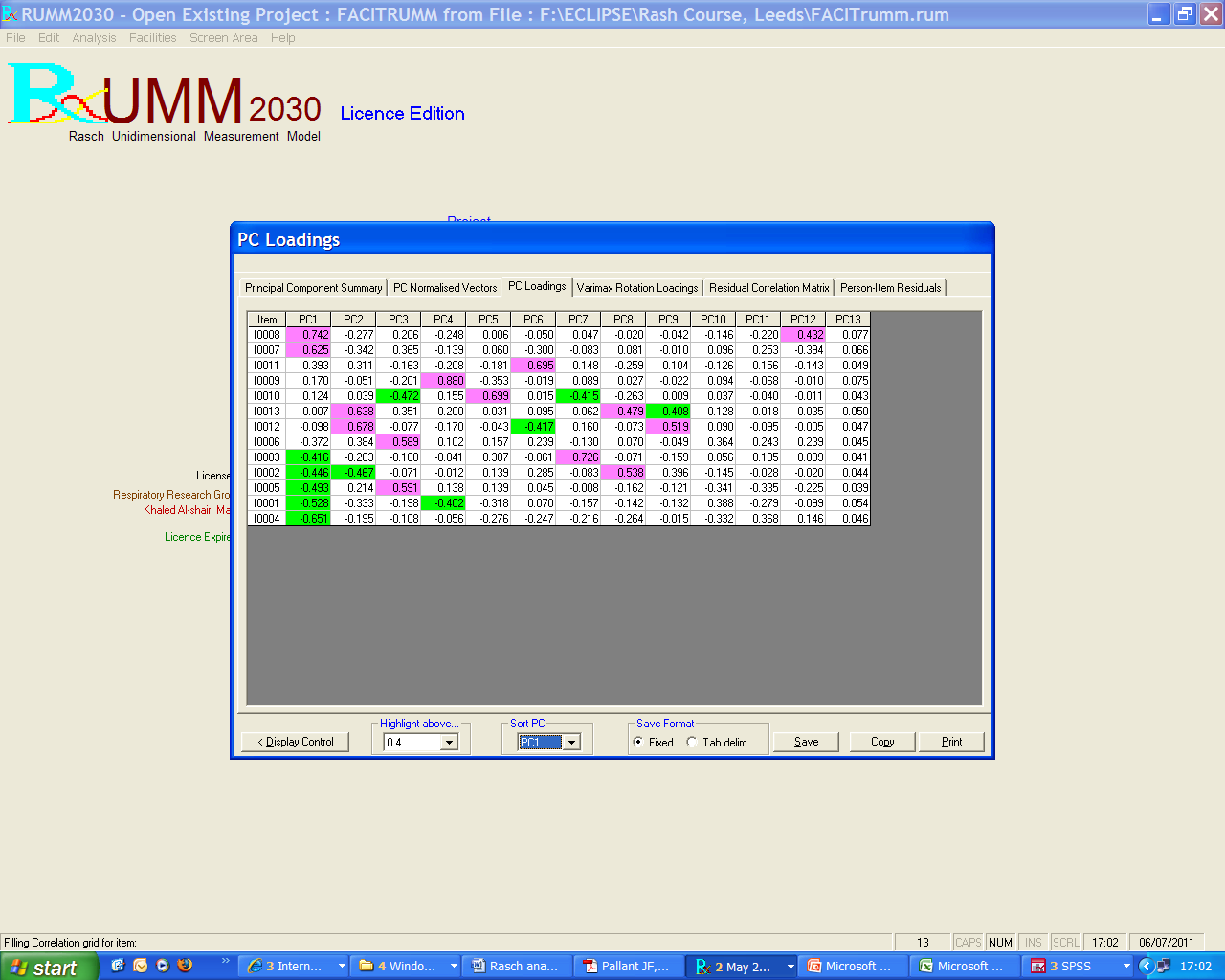
**

**Figure 4, components of the FACIT-F scale**


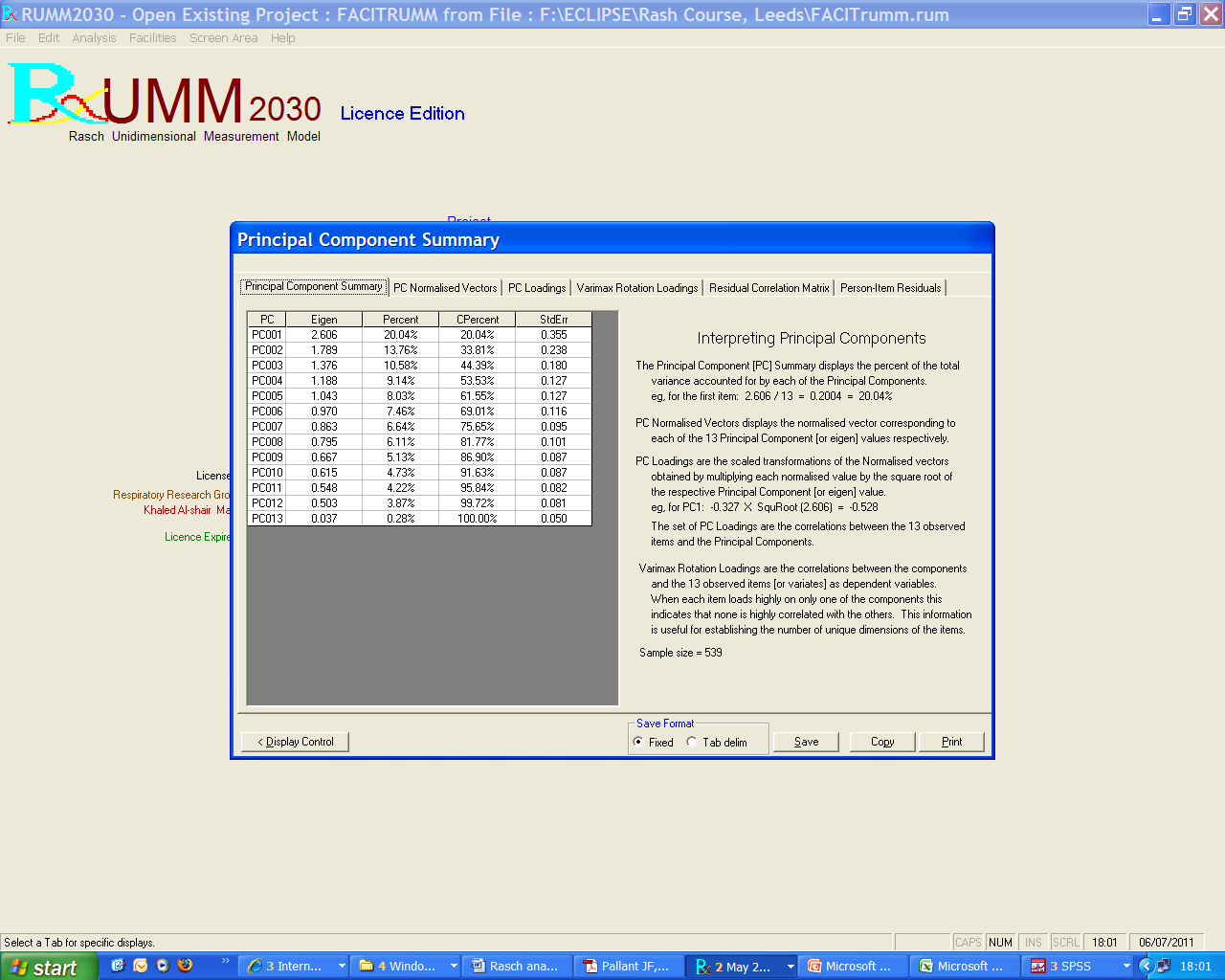


**References:**

CONAGHAN, P. G., EMERTON, M. & TENNANT, A. (2007) Internal construct validity of the Oxford Knee Scale: evidence from Rasch measurement. *Arthritis Rheum,* 57**,** 1363-7.

MILLS, R. J., YOUNG, C. A., PALLANT, J. F. & TENNANT, A. (2010) Rasch analysis of the Modified Fatigue Impact Scale (MFIS) in multiple sclerosis. *J Neurol Neurosurg Psychiatry,* 81**,** 1049-51.

PALLANT, J. F. & TENNANT, A. (2007) An introduction to the Rasch measurement model: an example using the Hospital Anxiety and Depression Scale (HADS). *Br J Clin Psychol,* 46**,** 1-18.

YORKE, J., JONES, P. W. & SWIGRIS, J. J. (2011) Development and validity testing of an IPF-specific version of the St George's Respiratory Questionnaire. *Thorax,* 65**,** 921-6.
